# Normal Face Detection Over a Range of Luminance Contrasts in Adolescents With Autism Spectrum Disorder

**DOI:** 10.3389/fpsyg.2021.667359

**Published:** 2021-07-16

**Authors:** Daniel J. Norton, Ryan K. McBain, Grace E. Murray, Juna Khang, Ziqing Zong, Hannah R. Bollacke, Stephen Maher, Deborah L. Levy, Dost Ongur, Yue Chen

**Affiliations:** ^1^McLean Hospital, Department of Psychiatry, Harvard Medical School, Belmont, MA, United States; ^2^Department of Psychology, Williams College, Williamstown, MA, United States; ^3^Gordon College, Wenham, MA, United States; ^4^RAND Corporation, Boston, MA, United States; ^5^Quality Metric, Johnston, RI, United States

**Keywords:** neurotypical, psychophysical, cognitive, recognition, perception

## Abstract

Face recognition is impaired in autism spectrum disorders (ASDs), but the reason for this remains unclear. One possibility is that impairments in the ability to visually detect faces might be a factor. As a preliminary study in this vein, we measured face detection ability as a function of visual contrast level in 13 individuals with ASD, aged 13–18, and 18 neurotypical controls (NCs) in the same age range. We also measured contrast sensitivity, using sinusoidal grating stimuli, as a control task. Individuals with ASD did not differ from controls in face detection (*p* > 0.9) or contrast detection (*p* > 0.2) ability. Performance on contrast and face detection was significantly correlated in ASD but not in NC. Results suggest that the ability to visually detect faces is not altered in ASD overall, but that alterations in basic visual processing may affect face detection ability in some individuals with ASD.

## Introduction

Many studies have examined various aspects of face processing in autism spectrum disorder (ASD), including identity recognition, discrimination between parts of faces or their configuration, and face emotion recognition (FER). The results of these studies are mixed. Some aspects of face processing, such as FER, appear to be impaired in at least some conditions (Jemel et al., 2006; Uljarevic and Hamilton, 2013; Lozier et al., 2014) and are related to social functioning (Trevisan and Birmingham, 2016). Others, such as perceiving the identity of a face, appear to be impaired overall in ASD, but not in all studies (Weigelt et al., 2012; Hadad et al., 2019; Griffin et al., 2021).

In general, seeing faces comprises several functional components, including recognizing the identity, gender, or emotional expression associated with a face image (Bruce and Young, 1986). The first step in this information-processing stream, distinct from downstream components like identity discrimination and FER (Damasio, 1985), is the detection of a face as such, or “face detection” (Purcell and Stewart, 1988; Garrido et al., 2008; Bindemann and Burton, 2009; Bindemann and Lewis, 2013). Face detection is presumably required in order to harness the specialized neural machinery that exists for other aspects of face recognition such as processing identity, emotional state, or other facial attributes (Bodamer, 1947; Kanwisher et al., 1997, 1998; Ishai et al., 1999; Tarr and Gauthier, 2000).

Individuals who cannot detect faces well are at a disadvantage for downstream face processing including FER and face identity discrimination for two reasons. First, individuals with poor face detection ability cannot take advantage of this specialized machinery as quickly or as often (Chen et al., 2008). In a more extreme scenario, individuals who fail to detect some faces in their environment would be precluded from appropriate social responses to those faces. A previous study speculated that individuals with ASD may be less likely to elicit face-specific event-related potentials to non-face objects than neurotypically developing controls (Churches et al., 2012), suggesting a narrowing of the range of stimuli to which the face processing system is activated in ASD.

Face detection has not been well characterized in ASD. One previous study used a visual search task to measure how quickly individuals could identify a face within a matrix of other faces, or of non-face objects (Moore et al., 2016). It found no difference in reaction time to locate a face between NC and individuals with ASD. Because this study was a visual search task, visual scanning strategies may have influenced performance; in addition, the face stimuli were presumably easy to perceive once they were fixated. Therefore, a key question remains as to whether individuals with ASD have intact ability to detect a face, when scanning through the image is not a major part of the task. In order to measure the integrity of low-level visual processing involved in face detection, apart from attentional processes involved in directing visual search, one would have to come up with a sufficiently difficult task that produces some variance in performance (e.g., Chapman and Chapman, 1973), without the need for a complex visual search to do the task.

In the present study, we performed a preliminary examination of face detection performance as a function of luminance contrast in a group of adolescent individuals with ASD and a comparison group of NC. The task used a brief presentation (~200 msec) which precludes extensive visual scanning (for further rationale on the specific face detection stimulus used in the present study, see Section “Procedure”). Prior work by our group has shown that this method provides a characteristic psychometric function in healthy and clinical populations (Maher et al., 2016). We hypothesized that individuals with ASD would perform worse than NC on face detection (Churches et al., 2012). We also measured performance on a control task—contrast detection, where participants detected a simpler visual object (a sinusoidal grating), to explore whether any deficits in face detection might be explained by more basic visual processing deficiencies. Prior work had shown mixed results in terms of individuals with ASD on contrast detection and other basic visual abilities such as motion processing (e.g., Spencer et al., 2000; Chen et al., 2012; Kéïta et al., 2014; Wright et al., 2014; Guy et al., 2016).

## Materials and Methods

### Subjects

Individuals with ASD were recruited from McLean Hospital, by online ads, and through ASD support groups in the Greater Boston area. Diagnoses were based on a standardized observation and interview using the Autism Diagnostic Observation Schedule (ADOS; Lord et al., 2000), conducted by clinicians who were trained for research reliability, and by a review of all available medical records. Of the 24 adolescents who were screened on the ADOS, 14 subjects met the threshold for an ASD (a score of 8 or higher on sections A and B). One of these subjects was excluded for performing at chance level, explained in further detail under the Section “Missing and Invalid Data” in the Supplementary Material. The resulting sample size for ASD, excluding this subject, was 13.

Eighteen healthy controls recruited from the same local communities also participated. They were screened for the absence of Axis I psychiatric disorders using a standardized interview based on the SCID-I/NP (First et al., 2002). General inclusion criteria for all participants were: (1) no history of neurological disorder (e.g., seizure, stroke) or head injuries, (2) verbal IQ ≥ 70 (for subjects older than 16 years and 9 months of age, the Wechsler Adult Intelligence Scale-Revised was used; Wechsler, 1981); for younger subjects, the Wechsler Intelligence Scale for Children (WISC) was used (Wechsler and Psychological Corporation, 1949), (3) age between 13 and 18 years old, (4) no self-reported substance abuse in the 6 months prior to participation, and (5) no diagnosis of ADHD and no current use of stimulant medication. Clinical and demographic information is listed in Table 1. The two groups of participants did not differ in terms of age *t*(25.7) = 0.90, *p* = 0.37, but differed significantly in verbal IQ, *t*(26.0) = 2.41, *p* = 0.02 (both *t*-tests with unequal variances assumed, hence adjusted degrees of freedom). All participants had normal or corrected-to-normal vision, as assessed by the Rosenbaum Pocket Vision Screener (Loewenfeld and Lowenstein, 1999).

**Table 1:** Demographics of the sample.

Group	Sex	Ethnicity	Age (year)	IQ^a^
NC (*n* = 18)	M-13	3 African American	15.7 (1.9)	106.4 (15.6)
	F-5	1 Asian		
		1 Hispanic		
		13 White		
ASD (*n* = 13)	M-10	13 White	15.7 (1.7)	97.1 (15.4)
	F-3			

Group mean (standard deviation). F, female; M, male. Subjects whose data were invalid and excluded from all analyses are not shown in this table (see Section “Missing and Invalid Data”).

a Based on The Wechsler Intelligence Scale-Child Version for individuals less than 16 years, 9 mo. Based on Wechsler Adult Intelligence Scale-Revised for individuals older than 16 years 9 mo.

All participants, accompanied by adult guardians, were briefed about the purpose of the study and subsequently signed assent forms. Guardians signed consent forms. The study protocol was approved by the Institutional Review Board of McLean Hospital.

### Procedure

After completing the demographic and clinical assessments, as well as the verbal IQ measures outlined above, subjects performed the face detection and contrast detection tasks. Each task took approximately 10 min, including instructions and a demonstration. There was opportunity for a break in between the tasks.

#### Face Detection

**Figure 1:**
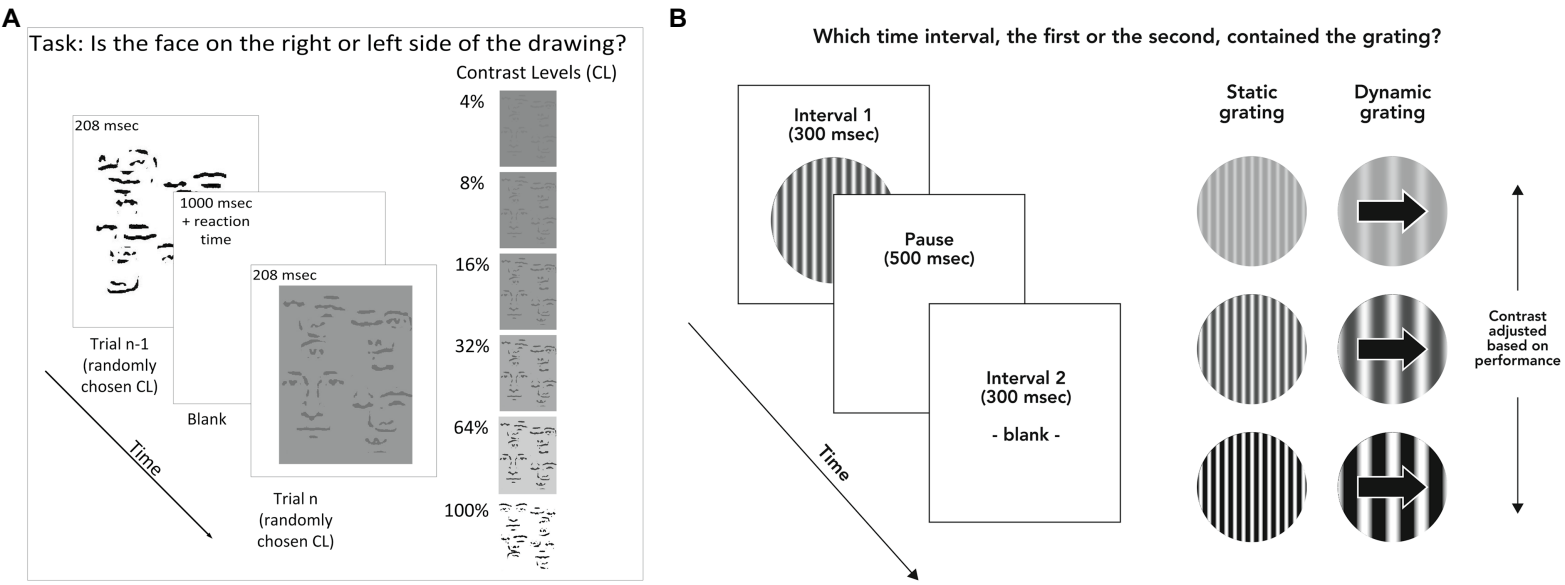
**(A)** Stimulus and task for face discrimination. Stimulus duration was 208 msec, with a pause of 1,000 msec between each trial to ensure participants had time to fixate. Participants responded by pressing arrow keys to indicate whether the face was on the left or right side of the stimulus. Stimuli were displayed at randomly varying contrast levels of 4, 8, 16, 32, 64, and 100% contrast. **(B)** Task and stimuli for contrast detection. Subjects indicated which of two time intervals (300 msec each) contained the stimulus (randomly determined each trial). In this example, the grating is shown in the first time interval. Each interval was accompanied by an auditory tone. Participants indicated whether the grating was presented during the first or second time interval by pressing the “1” or “2” key on a computer keyboard. Contrast levels during the task were adjusted according to performance on previous trials.

The face detection task measured the ability to recognize briefly presented line-drawn faces (illustrated in Figure 1A). We chose line-drawn faces for two reasons. By stripping the face images down to their simplest elements, we were able to use tilted and shifted copies of these elements as the noise, or non-face portion of the stimulus. This meant that the face portion of the stimulus contained features identical to the non-face (i.e., distracting area) portion of the stimulus, with the sole exception of their configuration. This choice of stimulus therefore removed the possibility that subjects would be able to identify a face based on local features such as skin texture, color, and hair shape. In addition, these stimuli show a strong facial inversion effect, suggesting that they possess elements of what makes face processing unique compared with other forms of object recognition (Garrido et al., 2008).

The faces were constructed by transforming photographed faces. These photographs were taken with overhead lighting, and then blurred, high-pass filtered, and thresholded (all in Adobe Photoshop), resulting in a face constructed of black lines on a white background (Garrido et al., 2008). The task consisted of one block of testing, which took about 15 min, including a demo to ensure that subjects understood the task and any breaks in between trials, which were allowed at will. The task was to determine which side of a scrambled line drawing, left or right, contained a line drawn face. The stimulus was presented for 208 msec. Four-line drawn faces and six contrast levels were used. Each condition was repeated eight times, for a total of 192 trials. This resulted in 16 trials per contrast level averaged across the four faces; accuracy across these trials served as the main outcome measure. The contrast level that corresponded to each subject’s perceptual threshold was calculated using a Weibull equation (see Supplementary Material for details), which allowed comparison of face detection task performance with other variables (Norton et al., 2009). Treatment of missing and invalid data is detailed in the Supplementary Material.

#### Contrast Detection

The contrast detection task was to determine which of two temporal intervals, each denoted by a different auditory tone, was associated with the presentation of a sinusoidal grating that subtended 10 degrees of visual angle across a circular window (Figure 1B). This task was chosen in order to rule out the possibility that deficiency in contrast sensitivity might account for a face detection deficit, since the face detection stimuli were defined by luminance contrast. There were two test blocks of about 4 min each.

The experimental paradigms for contrast detection and face detection were programmed in C and presented using the VisionShell software (Comtois) on Macintosh OS environments. The contrast detection task was presented on a 14" cathode ray tube Macintosh monitor equipped with an attenuator to allow precise control of contrast. For contrast detection, the instructions to subjects were as follows: “You’re going to hear two beeps. During one of the beeps, you will see some wavy lines come up onto the screen, and during the other beep you will not see anything. Your job is to tell me during which beep you saw the wavy lines, first or second.” The task terminated after 12 reversals of staircase direction (Chen et al., 2003). The threshold was defined as the mean of the contrast values at all reversals except the first and was the sole outcome measure for the task. The starting contrast for the dynamic (0.5 cpd, 5 Hz) grating was 1.5%, and for the static (4 cpd, 0 Hz) it was 2%. Increases and decreases in contrast after each trial were always set to 5% of the previous trial’s contrast value. For example, 5% decrease from the initial 1.5% contrast would result in a contrast of 1.5% × 0.95, or 1.425% contrast.

Each block of trials used a 3-down 1-up staircase procedure, where three correct responses in a row caused the contrast of the next stimulus to decrease one incorrect trial caused it to increase. See Supplementary Material for further detail.

There were two task conditions for grating contrast detection. The first, referred to as the dynamic grating condition, used a spatial frequency (SF) of 0.5 cycles per degree of visual angle (cpd) and a temporal frequency of 5 Hz. Spatial frequency refers to the frequency over space with which a pattern repeats itself. The second, referred to as the static condition, used a spatial frequency of 4 cpd and a temporal frequency of 0 Hz. These conditions were chosen in order to tap neural processes thought to be associated with the parvocellular and magnocellular systems (Skottun, 2000; Kim et al., 2006). We anticipated that the static condition would be more closely related to face detection due to its closer match to the face detection stimuli in terms of spatial frequency and static position over time. For the analyses, the logarithm of raw threshold score from each condition was used as the primary outcome measure, as is the standard for these types of data (Pelli and Bex, 2013). Group differences in contrast detection at each of the two frequencies were analyzed using mixed model ANOVAs with spatial frequency as the within-subjects factor and diagnostic group as the between-groups factor. Further detail on the contrast detection stimulus and procedure is included in the Supplementary Material.

### Missing and Invalid Data

One subject with ASD responded by pressing the same button on each trial and achieved 50% accuracy on every face, at every contrast level. This subject was excluded from all analyses on the assumption that he did not understand the task and his responses were not a valid reflection of his ability to detect faces. Two additional subjects with ASD and two control subjects were missing contrast detection data, resulting in sample sizes of 11 and 16 subjects, respectively, in calculating correlations between contrast and face detection scores. One NC subject did not receive an assessment of verbal IQ, so the size of the NC sample for correlations involving this variable was 17. Finally, one NC subject was missing data on the two highest contrast conditions for all four faces. His missing data for these conditions were replaced using the mean of 5 multiple imputations based on a regression equation considering subject group, contrast detection thresholds, age, verbal IQ, and the accuracies from the lower contrast face conditions (Schafer, 1999).

## Results

### Face Detection

**Figure 2:**
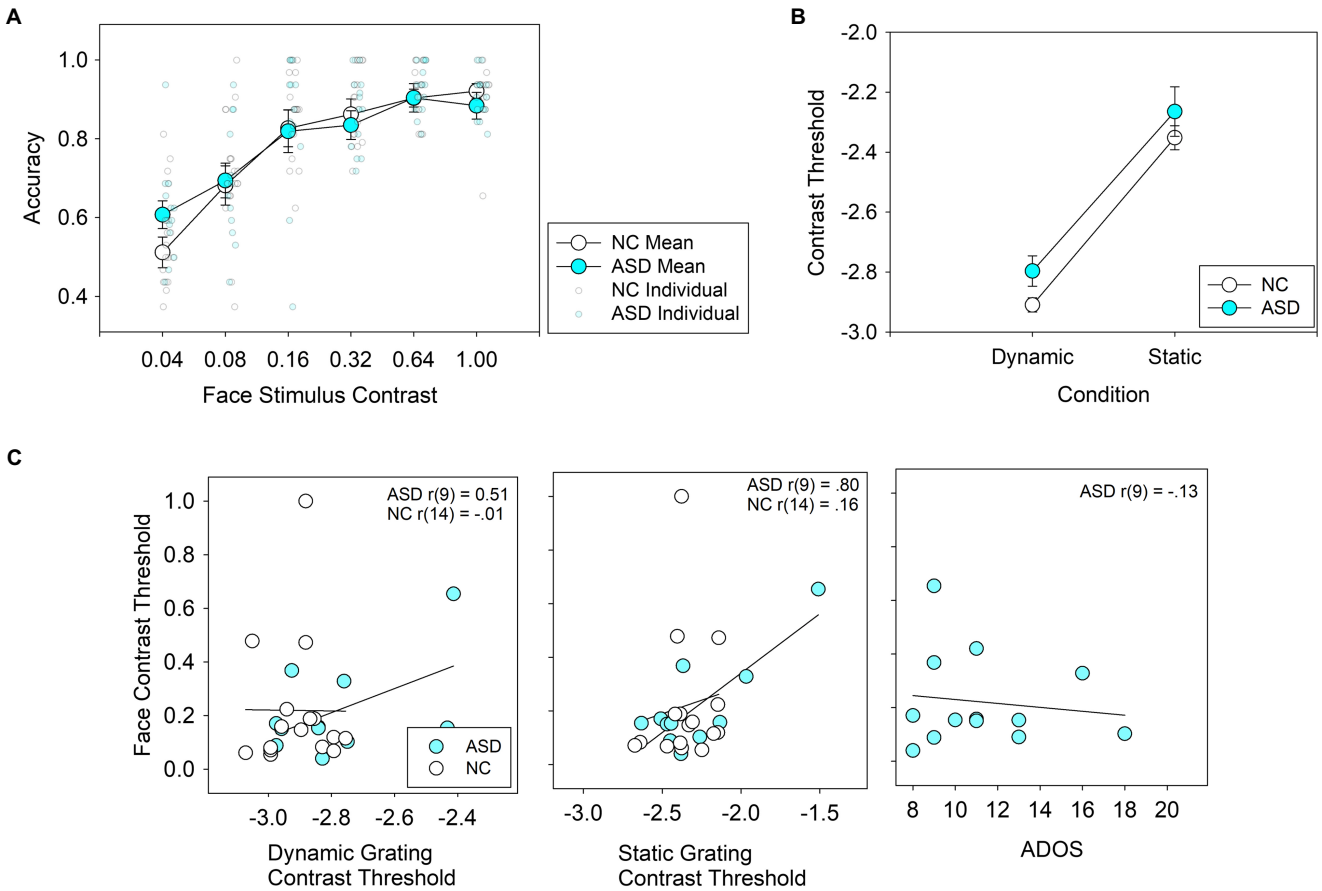
**(A)** Average accuracy for face detection in individuals with autism spectrum disorder (ASD) and neurotypical controls (NC) across 6 contrast levels. Group means are represented by large, bold circles, and individual scores are represented by semi-translucent, small circles. **(B)** Average thresholds for contrast detection using grating stimuli. **(C)** Correlations between face detection thresholds and dynamic contrast detection thresholds (left panel), static contrast detection thresholds (middle panel) and ADOS scores (right panel).For the left and middle panels, the logarithm of grating contrast detection thresholds is shown.

Averaged performance across the four face stimuli used in the face detection task is shown in Figure 2A; performance for each face separately is shown in the Supplementary Material (Supplementary Figure S1). A three-way ANOVA with face stimulus (one of four), contrast level, and group as factors showed main effects for contrast level, *F*(5,145) = 67.1, *p* < 0.001, *η*^2^ = 0.70, and face stimulus, *F*(3,87) = 23.4, *p* < 0.001, *η*^2^ = 0.45, but not for group, *F*(1,29) = 0.00, *p* = 0.96, *η*^2^ = 0.00. There were no significant interactions between group and contrast, *F*(5,29) = 1.12, *p* = 0.35, *η*^2^ = 0.04 or group and face stimulus, *F*(3,29) = 0.62, *p* = 0.61, *η*^2^ = 0.02, or group × contrast × face stimulus *F*(15,29) = 1.41, *p* = 0.14, *η*^2^ = 0.05.

Averaged reaction times for each of the four face stimuli used in the face detection task are shown in Supplementary Figure S2. A three-way ANOVA with face stimulus (one of four), contrast level, and group as factors showed main effects for contrast level, *F*(5,145) = 9.65, *p* < 0.001, *η*^2^ = 0.26, and face stimulus, *F*(3,135) = 3.1, *p* = 0.031, *η*^2^ = 0.10, but not for group, *F*(1,29) = 0.03, *p* = 0.86, *η*^2^ = 0.00. There were no significant interactions between group and contrast, *F*(5,29) = 0.74, *p* = 0.59, *η*^2^ = 0.03 or group and face stimulus, *F*(3,29) = 1.45, *p* = 0.23, *η*^2^ = 0.05, or group × contrast × face stimulus *F*(15,29) = 0.94, *p* = 0.52, *η*^2^ = 0.03.

A Bayesian ANOVA was performed using the JASP software (Rouder et al., 2012; Morey and Rouder, 2015). The ANOVA was set up as the classical ANOVA was, using a 4 (faces; within subjects) × 6 (contrast level; within subjects) by 2 group (ASD, HC; between subjects) design. JASP tested every combination of factors, assigning equal probability to each possible model given the design (*p*(*M*) = 1/19 = 0.053 for each possible model). The model that best explained the data was the within subject design (Face + Contrast + Face×Contrast) see Supplementary Table S1. The next best model simply added the group factor, which resulted in worse performance, indicating that ASD vs. NC was not an informative variable in determining performance. Overall, this analysis reveals similar results to the original classical ANOVA results.

### Contrast Detection

Performance on the contrast detection task in ASD subjects is shown in Figure 2B. ANOVA revealed a main effect for stimulus type (low spatial frequency-dynamic vs. high spatial frequency-static), *F*(1,25) = 149.1, *p* < 0.001, *η*^2^ = 0.86, but not for group, *F*(1,25) = 2.19, *p* = 0.15, *η*^2^ = 0.08. The interaction between group × stimulus type, *F*(1,25) = 0.34, *p* = 0.57, *η*^2^ = 0.01, was not statistically significant.

### Correlation Between Face Detection and Other Variables

Correlations between face detection thresholds and IQ were not statistically significant in ASD: *r*(11) = 0.10, *p* = 0.75, or in NC *r*(15) = 0.019, *p* = 0.94. The correlations between face detection thresholds and contrast thresholds and ADOS scores (for the ASD group only) are shown in Figure 2C. In the control group, face detection thresholds were not correlated with contrast detection thresholds in the dynamic, *r*(14) = −0.014, *p* = 0.96, or static condition, *r*(14) = 0.155, *p* = 0.55. In the ASD group, face detection thresholds were correlated with contrast detection thresholds in the static condition, *r*(9) = 0.80, *p* = 0.003, but not significantly so in the dynamic condition, *r*(9) = −0.47, *p* = 0.14. In the ASD group, ADOS scores did not correlate significantly with face detection thresholds, *r*(9) = −0.13, *p* = 0.67.

## Discussion

Our results suggest that face detection *per se* is not impaired in ASD, a novel finding in the ASD face processing literature. No statistically significant differences in accuracy distinguished the groups, nor were there any interactions between group and face stimulus or contrast level. The results suggest that deficits in ASD regarding downstream face processing, such as recognition of identity, age, gender, and emotion may emerge after this initial phase of face detection.

The present study employed several strategies that were advantageous. First, it used a two-alternative forced choice paradigm (“Which side was the face on?”), which removed subjective criterion bias, which is a factor in other methods, such as “Did you see the face?,” Yes/No. In addition, the use of line-drawn face stimuli, which were stripped of cues about identity, age, and gender, removed any higher-order face processing interferences as confounders. Rather, the strategy used here measured performance as a function of stimulus contrast, providing an unbiased picture of individuals’ basic perceptual abilities. Further, it used briefly presented faces (208 msec), which reduced the degree to which eye scanning patterns might confound performance. These considerations strengthen the interpretation that our results reflect face detection perceptual ability rather than higher-order cognitive or emotional factors.

Despite the absence of a group difference in face detection accuracy, performance on that task was significantly correlated with contrast detection in the ASD subjects only. This correlation was especially strong for the static contrast detection condition, which explained over 60% of the variance in face detection thresholds. The preferentially strong relation to face detection in the static condition may be due to the similarities in spatial and temporal frequency between the face and grating stimuli in that condition (i.e., fairly high spatial frequency, not moving). The correlation may have been detected only in ASD because of the larger variability and range in their contrast detection thresholds than in NC. The few ASD who had very high thresholds on the static condition did poorly on face detection as well, possibly due to an inability to make out the lines comprising the face stimulus on the low contrast conditions at that spatial frequency. Given the small sample size, this correlation result should be interpreted with great caution and requires replication in a larger sample. Notably, the correlation between static contrast detection and face detection is no longer statistically significant in the ASD group when the subject with the highest face detection threshold and contrast detection threshold was removed. Although we did not find a group difference for contrast detection between ASD and NC, the use of an auditory tone to denote time intervals may have affected performance on the task, specifically within ASD, which often presents with differences in auditory processing.

A prior study showed that individuals with ASD show an advantage on identifying faces that were filtered to contain only high SF information, as compared to faces that were filtered to contain only low SF information (Deruelle et al., 2004). One possible reason for that result is that individuals with ASD preferentially rely on high SF information when viewing faces in general. Such a preferential reliance on higher SF information to process faces would be consistent with face detection performance in ASD being largely determined by ability to see low-level visual features in that spatial frequency range, as shown in the present study. Of course, with the small sample size and exploratory nature of this study, the finding requires replication and the explanation offered is tentative. It is also interesting to note that the ability to detect a face when consciously attempting to do so is distinct from the degree to which brain mechanisms may naturally draw one’s attention toward a face in a natural setting. Though the former was shown to be intact in the present study, the latter was not.

### Limitations and Future Directions

The present study had several limitations, including a small sample, underscoring the need for replication. Notably though, none of the effect sizes for group comparisons approached the alpha cutoff level of 0.05, making it unlikely that low power is the basis for the absence of group differences. Also, our sample included only relatively high functioning ASD individuals from ages 13 to 18; whether this finding generalizes to lower functioning individuals with ASD, or to other age ranges requires additional study. Further, we used the WAIS-R (Wechsler, 1981) and the WISC (Wechsler and Psychological Corporation, 1949) to estimate verbal IQ, which are not comparable to current norms, though they do serve the purpose of showing that our sample of ASD subjects likely had a lower verbal IQ than the NC subjects. Another limitation was the lack of non-verbal IQ estimates in the present study. Finally, there are a number of other comparisons that might be have been useful to make with face detection data, including eye tracking data, electrophysiology, or other clinical measures. One particularly interesting comparison would have been a FER task, which was not administered in the present study. The literature generally implicates FER problems in ASD (Uljarevic and Hamilton, 2013; Lozier et al., 2014), and our study suggests that face detection *per se* is unlikely to account for FER deficits, but this conclusion is speculative at this point given the lack of direct comparison in our sample. There was also considerable variability in face detection ability in the ASD group, so we cannot exclude the possibility that only a subgroup of individuals with ASD has impaired face detection ability. Examining face detection performance in a larger sample, and comparing it to FER performance in the same group of subjects, would be an interesting line of future research.

## Data Availability Statement

The raw data supporting the conclusions of this article will be made available by the authors, without undue reservation.

## Ethics Statement

The studies involving human participants were reviewed and approved by McLean Hospital IRB. Written informed consent to participate in this study was provided by the participants’ legal guardian/next of kin.

## Author Contributions

DN, RM, and YC were involved with study design. DN and RM collected the data. DN, RM, SM, and YC analyzed the data. All authors were involved in interpretation of the data and editing the manuscript. DN drafted the manuscript. All authors contributed to the article and approved the submitted version.

### Conflict of Interest

Author SM was employed by the company Quality Metric.

The remaining authors declare that the research was conducted in the absence of any commercial or financial relationships that could be construed as a potential conflict of interest.
