# Supplementary material for: Normal Face Detection Over a Range of Luminance Contrasts in Adolescents With Autism Spectrum Disorder
Source: Front Psychol. 2021 Jul 16;12:667359. doi: 10.3389/fpsyg.2021.667359 (PMC8322772; doi:10.3389/fpsyg.2021.667359)
Supplement: Supplementary file 1 [file Data_Sheet_1.PDF]

Appendix

Methods

Face Detection:

The display for the face stimuli was a 20" Macintosh LCD monitor with a refresh rate of 60 Hz. The contrast values presented were calculated using the standard Michelson formula, $c = \frac{(\max - \min)}{(\max + \min)}$ but instead of max and min representing lumanance values, they simply represent the RGB values sent to the monitor (which ranged from 0-255). The face stimulus was presented at 265 x 355 pixels subtending 13 x 17 degrees of visual angle. The instructions were as follows: 'You're going to see a bunch of squiggly lines come up on the screen, and somewhere in there, there is going to be a face. The face will be on the left or the right, and there will always be one present. Your job for each trial is to tell me whether the face was on the left or right. If you can't tell, just take your best guess".

To calculate contrast thresholds for the face detection task, raw accuracy values, averaged across the four face stimuli, were calculated for each contrast level, and fit to the Weibul equation $y = 1 - 0.5 * e^{(\frac{-x}{a})^{b}}$, where *y* is the proportion correct, *x* is the speed of rotation, and *a* and *b* are curve fitting parameters. Specifically, *a* denotes the point at which the function begins to rise from its lower asymptote, and *b* denotes the slope of the rising portion, with smaller values for b indicating steeper slopes. The threshold was considered to be the contrast value corresponding to 80% accuracy, as in previous work (Norton, McBain, Holt, Ongur, & Chen, 2009).

Analyses:

Group differences in accuracy were analyzed using a 3-way, mixed model ANOVA with contrast level and face stimulus (each of the four used as a different level) as the within-subjects factors and diagnosis as the between-groups factor.

For each subject group, Pearson correlations were analyzed between each participant's face detection threshold and verbal IQ, as well as between the face detection threshold and the logarithm of the contrast detection thresholds at both frequencies. For ASD participants, a Pearson correlation was calculated between each participant's face detection threshold and total ADOS score.

Results

Group means for accuracy in each of the four face stimuli used are shown in figure S1, while means for reaction time are shown in figure s2. The mean of these four face stimuli at each contrast level are represented in Figure 2a in the main text. The detailed results of the Bayesian analysis reported in the main text are shown in table S1, generated in JASP (JASP team, 2018).Overall, this analysis reveals similar results to the original classical ANOVA results.

Discussion:

Many prior studies have examined FER in ASD, and have generally found deficient performance in ASD (14). Our results suggest that an impaired ability to detect faces is not a primary contributor to the FER deficit. Seen another way, social cognition deficits in individuals

with ASD—including FER—may be driven by higher-order, or parallel processes, or by perceptual impairments that only become vulnerabilities when higher order face processing, e.g., detecting subtle shifts in arrangement of visual features, is required. By contrast, in schizophrenia low-level perceptual processes, such as contrast detection and face detection, are implicated in FER deficits (Chen et al., 2008; Norton, McBain, Holt, et al., 2009); in ASD, this appears not to be the case. This finding could lead to further research to more explicitly differentiate the components of FER deficits in clinical populations, and ultimately increase our understanding of the underlying processes that lead to impaired social cognition.

Reference:

Chen, Y., Norton, D., Ongur, D., & Heckers, S. (2008). Inefficient face detection in schizophrenia. *Schizophrenia Bulletin*, *34*(2), 367–374. https://doi.org/10.1093/schbul/sbm071

Comtois, R. (2003). Vision Shell Software. *Cambridge*, MA.

JASP Team (2018). JASP (Version 0.8.6) [Computer software].

Morey, R. D., & Rouder, J. N. (2015). BayesFactor (Version 0.9.11-3)[Computer software].

Norton, D., McBain, R., Holt, D. J., Ongur, D., & Chen, Y. (2009). Association of impaired facial affect recognition with basic facial and visual processing deficits in schizophrenia.

Biological Psychiatry, *65*(12), 1094–1098. https://doi.org/10.1016/j.biopsych.2009.01.026

Rouder, J. N., Morey, R. D., Speckman, P. L., & Province, J. M. (2012). Default Bayes factors for ANOVA designs. Journal of Mathematical Psychology, 56, 356-374.

Models	P(M)	P(M|data)	BF $_M$	Compared to best model		Compared to Null model	
				BF $_{10}$	error %	BF $_{10}$	error %
Contrast + Face + Contrast ✻ Face	0.053	0.833	89.902	1		4.826e +73	1.265
Contrast + Face + Contrast ✻ Face + Group	0.053	0.155	3.306	0.186	2.384	8.988e +72	2.02
Contrast + Face + Contrast ✻ Face + Group + Face ✻ Group	0.053	0.007	0.123	0.008	2.327	3.917e +71	1.953
Contrast + Face + Contrast ✻ Face + Group + Contrast ✻ Group	0.053	0.005	0.084	0.006	3.277	2.688e +71	3.023
Contrast + Face + Contrast ✻ Face + Group + Contrast ✻ Group + Face ✻ Group	0.053	2.107e -4	0.004	2.528e -4	2.528	1.220e +70	2.189
Contrast + Face + Contrast ✻ Face + Group + Contrast ✻ Group + Face ✻ Group + Contrast ✻ Face ✻ Group	0.053	3.216e -5	5.789e -4	3.860e -5	2.251	1.863e +69	1.861
Contrast + Face	0.053	1.314e -6	2.366e -5	1.578e -6	1.432	7.613e +67	0.671
Contrast + Face + Group	0.053	2.317e -7	4.170e -6	2.781e -7	1.86	1.342e +67	1.363
Contrast + Face + Group + Face ✻ Group	0.053	1.008e -8	1.815e -7	1.210e -8	4.139	5.841e +65	3.941
Contrast + Face + Group + Contrast ✻ Group	0.053	6.113e -9	1.100e -7	7.337e -9	2.511	3.541e +65	2.169
Contrast + Face + Group + Contrast ✻ Group + Face ✻ Group	0.053	2.468e -10	4.442e -9	2.962e -10	2.755	1.429e +64	2.447
Contrast	0.053	2.058e -22	3.704e -21	2.470e -22	1.478	1.192e +52	0.763
Contrast + Group	0.053	3.523e -23	6.342e -22	4.229e -23	1.881	2.041e +51	1.392
Contrast + Group + Contrast ✻ Group	0.053	7.272e -25	1.309e -23	8.727e -25	2.078	4.212e +49	1.648

Face	0.053	1.842e -64	3.315e -63	2.211e -64	1.564	1.067e +10	0.919
Face + Group	0.053	2.906e -65	5.231e -64	3.488e -65	1.822	1.683e +9	1.311
Face + Group + Face ✻ Group	0.053	8.777e -67	1.580e -65	1.053e -66	12.47	5.084e +7	12.402
Null model (incl. subject)	0.053	1.726e -74	3.108e -73	2.072e -74	1.265	1	
Group	0.053	2.697e -75	4.854e -74	3.237e -75	1.86	0.156	1.363

Table S1 Legend

P(M) - prior probability (i.e., probability of model before data)

P(M|data) - posterior probability, or probability or model given data (i.e., after considering data)

BF M - posterior model odds

BF 10 - Bayes Factor; comparison of given model to another model, usually the Best model (as defined by the greatest P[M|D]) or the Null model. Calculated as the (Best or Null[model average score across whole distribution + Error]) model posterior divided by each model. The result is reflects how many times better the Best/Null model is than the given model.

Figure S1

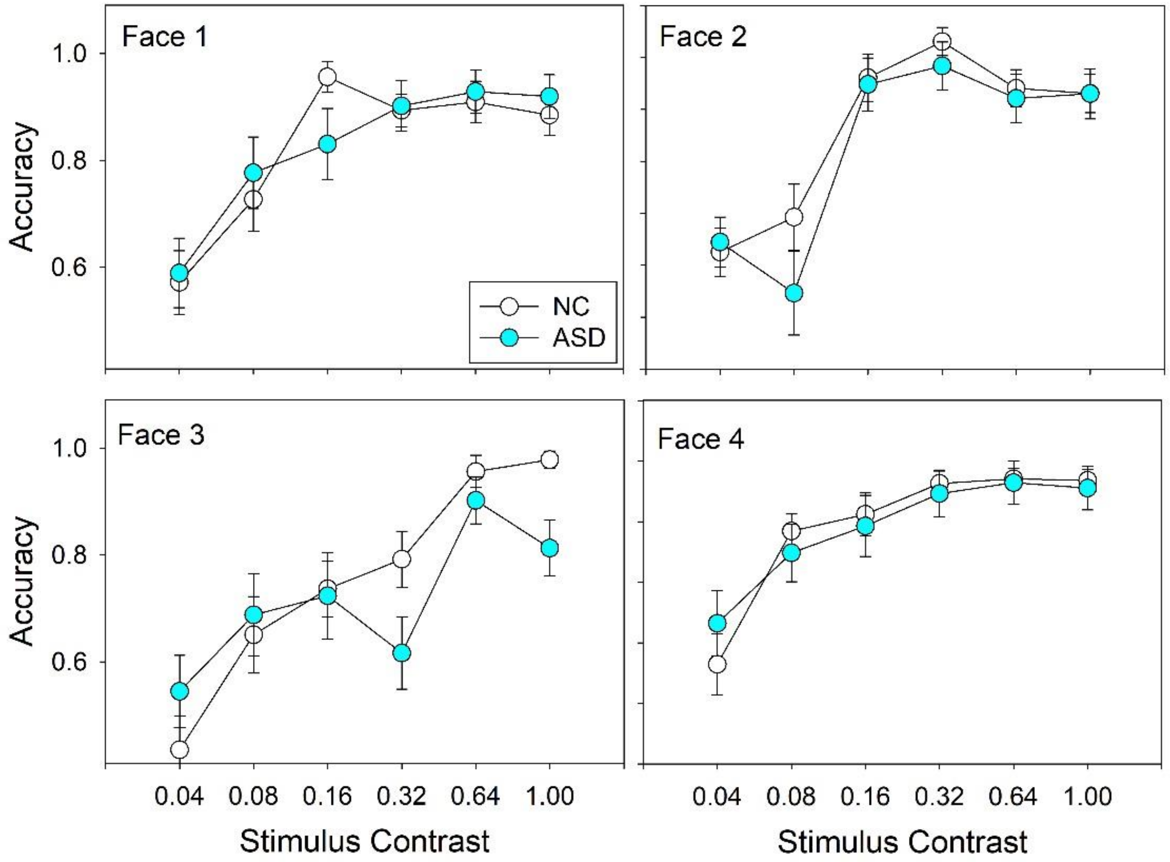

Figure S1 Legend

Accuracy on face detection as a function of stimulus contrast in individuals with autism spectrum disorder (ASD) and neurotypically developing controls (NC). Group means are shown for each of the four face stimuli used.

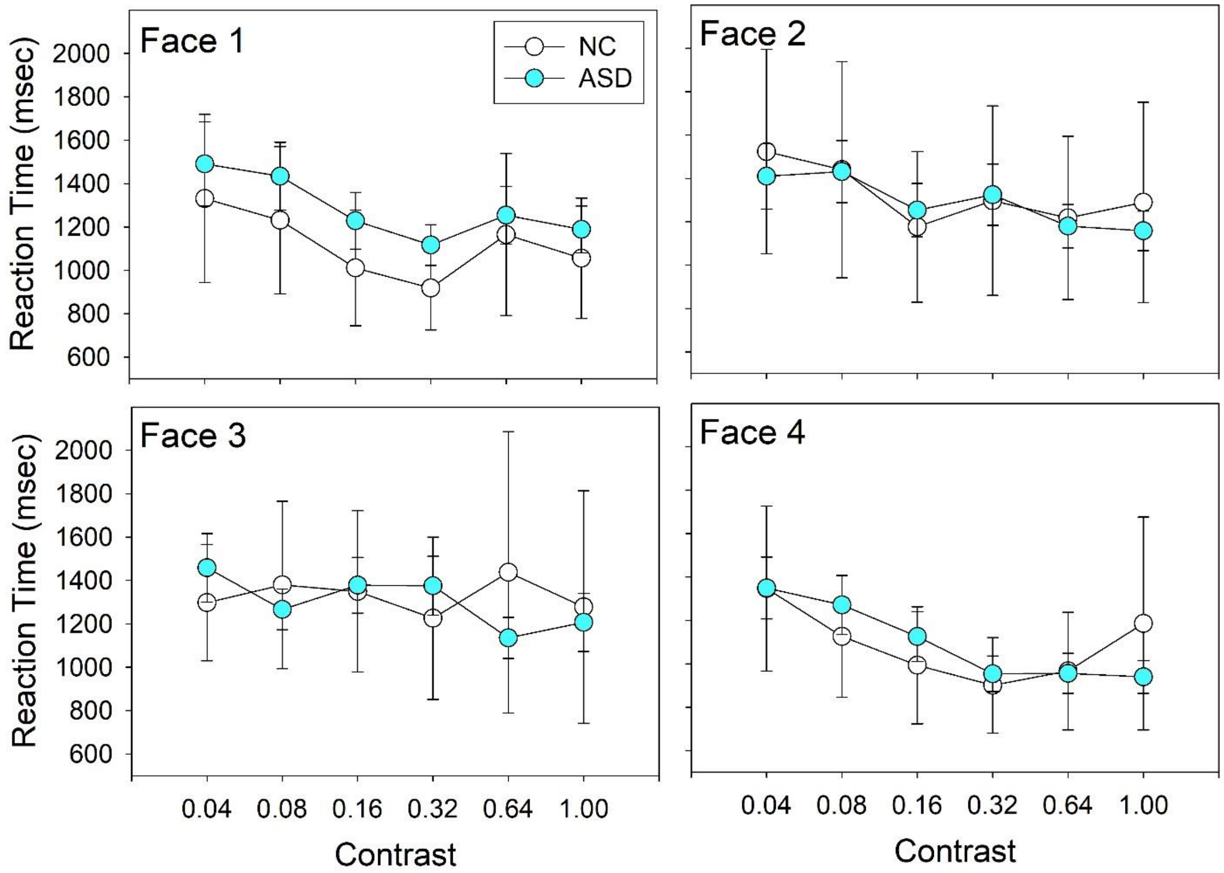

Figure S2 Legend

Reaction time on face detection as a function of stimulus contrast in individuals with autism spectrum disorder (ASD) and neurotypically developing controls (NC). Group means are shown for each of the four face stimuli used.
